# Letter to the editor: Autoimmunity in endometriosis: time to reframe our diagnostic and treatment approach

**DOI:** 10.1097/MS9.0000000000004066

**Published:** 2025-10-09

**Authors:** Aliya Noor, Akshay Lohana, Siddhant Kumar Yadav

**Affiliations:** a Department of Medicine, King Edward Medical University, Lahore, Punjab, Pakistan; b Department of Medicine, Liaquat University of Medical & Health Sciences, Jamshoro, Sindh, Pakistan; c Department of Medicine, Patan Academy of Health Sciences, Lagankhel, Lalitpur, Nepal

Endometriosis, a sinister disease that is often perceived as something solely related to pelvic organs, can virtually affect any part of the body. Endometriotic lesions have been identified in the lungs, diaphragm, pleura, pericardium, gastrointestinal tract, urinary tract, and even the central nervous system^[1]^. It begs the question whether endometriosis is just a gynecological condition or something more, an autoimmune disease? In accordance with TITAN 2025 guidelines, this manuscript guarantees transparency regarding the use of AI^[2]^.

For many years, it has been debated that endometriosis being an auto immune disease is not far from the truth. Previously, compiled evidence have demonstrated a great association between endometriosis and autoimmune diseases^[3]^. Many immunological studies reveal that endometriosis might be more of a systemic inflammatory disease rather than a gynecological condition. It is supplemented by the fact that women with endometriosis exhibit elevated levels of pro-inflammatory cytokines, altered T-cell ratios, and increased macrophage activity, which fits the profile of systemic inflammatory disease^[4]^.

A recent case control study showed that patients with endometriosis had a significantly higher risk of developing autoimmune conditions like rheumatoid arthritis, Hashimoto’s disease, systemic lupus erythematosus, multiple sclerosis, pernicious anemia, Sjögren’s syndrome, or myositis within 2 years of endometriosis diagnosis, which is a great breakthrough^[5]^. It has also been contemplated that endometriosis and autoimmune disease share the same genetic basis. This statement is supported by a recent retrospective cohort study, which suggests that endometriosis and autoimmune diseases share the same pathogenic pathways^[6]^.

Many studies done on gynecological disorders have elucidated that there is a delay of several years from symptoms to diagnosis of endometriosis, which has a significant impact on the quality of life. This is because endometriosis often presents with atypical symptoms and gets misdiagnosed due to non-specific diagnostic techniques^[7]^. The clinical implications of considering endometriosis as a potential autoimmune condition are huge. It shows that there is a possibility to diagnose endometriosis early by just screening the patients for autoimmune diseases.

The female patients who present with periodic atypical symptoms should undergo evaluation for autoimmune markers. Similarly, it puts patients with a family history of autoimmune disorders on the endometriosis radar. Immunomodulatory therapies could be developed for endometriosis, which is usually treated with hormonal and surgical therapies. Distinct immunological profiling for each patient can guide personalized treatment for each patient and improve outcomes.

Medical care provided to the endometriosis patients could be exponentially improved by coordination between gynecologists and immunologists. Endometriosis treatment can be improved by shifting the focus to identifying potential biomarkers for screening and evaluating specific immunomodulatory therapies in future clinical trials. The clinical guidelines regarding endometriosis diagnosis and treatment should be updated accordingly. Raising awareness about endometriosis as being a potential autoimmune disease, not just a simple gynecological disorder, would change the public and healthcare perspective. It would lead to an increase in the number of research studies and high-quality clinical trials done on the topic of endometriosis being an autoimmune disease, which will further improve patient care.

## Data Availability

Not applicable.
